# Hybrid semantic-lexical framework for ambiguity detection in software requirements using TF-IDF, character n-grams, and SBERT

**DOI:** 10.3389/frai.2026.1901190

**Published:** 2026-10-05

**Authors:** Fariha Khalid, Muhammad Yaseen, Muhammad Awais Sattar, Abdelhamid Ghoul, Aida Mustapha

**Affiliations:** 1 Faculty of Computing, Riphah International University, Lahore, Pakistan; 2 Automatic Control, Department of Computer Science, Electrical and Space Engineering, Luleå University of Technology, Luleå, Sweden; 3 Faculty of Computing, Universiti Malaysia Pahang Al-Sultan Abdullah, Pekan, Pahang, Malaysia

**Keywords:** ambiguity detection, hybrid feature representation, natural language processing, requirements engineering, Sentence-BERT (SBERT), software requirements specification, text classification

## Abstract

Ambiguity in software requirements is a major cause of misinterpretation, design defects, rework, and project delays in software engineering. This study proposes a hybrid semantic–lexical ambiguity detection framework that integrates lexical, structural, and contextual representations for identifying ambiguous software requirement statements. Two benchmark datasets were employed, including a Software Requirements Specification (SRS) dataset and a Functional–Non-Functional Requirements (FR–NFR) dataset. Data pre-processing involved tokenisation, lemmatisation, and noise removal, while Synthetic Minority Oversampling Technique (SMOTE) was applied to address class imbalance within training partitions. Three modeling strategies were evaluated: baseline classifiers using TF–IDF and character n-gram features, semantic models based on Sentence-BERT (SBERT) embeddings, and a hybrid representation combining TF–IDF, character n-grams, and SBERT embeddings. Experimental results indicate that baseline models achieved high precision but comparatively lower recall, limiting their ability to identify ambiguous requirements under class imbalance. The proposed hybrid representation achieved the strongest and most balanced performance across both datasets in practical terms, particularly when combined with Support Vector Machine classification, resulting in improved recall and F1-score, although statistical testing indicated that these gains over the strongest lexical baseline were not significant at the 5% level. By jointly modeling lexical vagueness, structural ambiguity patterns, and contextual semantics, the framework captures both explicit and implicit forms of ambiguity that are frequently overlooked by single-representation approaches. To strengthen experimental validity, five-fold stratified cross-validation, transformer-based baseline comparison using fine-tuned BERT, statistical significance testing, and annotation reliability assessment using Cohen's Kappa coefficient were incorporated. The findings demonstrate that integrating lexical, structural, and semantic representations provides a robust and potentially scalable approach for ambiguity detection in software requirements engineering. The proposed framework contributes to improved requirements quality, reduced risk of misinterpretation, and more reliable automated support for requirements analysis.

## 1 Introduction

The software development process requires a clear statement of user requirements and expectations of the system. These requirements outline what the system does and define its operational boundaries. They bridge the gap between users and developers to ensure that the intended functionality is correctly implemented. The requirements are normally expressed in natural language to be easily understood (Birhane, 2021). However, the use of natural language increases complexity and may introduce misunderstandings and development errors (Riaz et al., 2019).

Requirements engineering is therefore a major contributor to project success in software engineering. It is the stage at which user requirements are gathered, analyzed, and documented in the form of a Software Requirements Specification (SRS). The SRS specifies what the system is expected to accomplish and defines the quality attributes it must satisfy. It also functions as a formal agreement between stakeholders and system developers. A systematic and well-defined SRS minimizes ambiguity and provides a foundation for subsequent phases, including design, implementation, and testing. In contrast, unclear or poorly structured requirements frequently result in project delays, rework, increased cost, and low-quality software products (Kosenkov et al., 2024).

Ambiguity occurs when a requirement is open to more than one interpretation. Such ambiguity can confuse developers, testers, and clients, ultimately leading to systems that fail to meet user expectations. Ambiguous requirements affect project cost, delivery schedules, and overall software quality, and they create communication barriers among development teams and stakeholders (Veizaga et al., 2024; Aqsa et al., 2021). Ambiguity has been widely recognized as one of the primary causes of requirement-related problems, often arising from linguistic limitations, lack of contextual clarity, or vague terminology (Juhnke et al., 2021; Sonbol et al., 2022).

Early research attempted to address this problem through rule-based systems that scanned requirement text using pre-defined patterns or keyword lists. These approaches were relatively straightforward to implement and required limited computational resources. However, they lacked deeper conceptual understanding and were unable to identify implicit or context-dependent ambiguity. In addition, their adaptability across different document types and domains was limited (Mir Khatian et al., 2021). Machine learning techniques later improved ambiguity detection by learning statistical patterns from textual data represented through Bag-of-Words and TF-IDF features (Aqsa et al., 2021). Despite this improvement, such models largely operate at a surface lexical level and fail to capture sentence structure, contextual relationships, and semantic meaning.

Recent advances in natural language processing have introduced transformer-based architectures such as BERT and GPT, which generate contextual representations through large-scale pre-training (Ezzini et al., 2021). These models learn word meaning based on surrounding context and can be adapted to specialized domains through transfer learning. Transformer models have shown strong performance in tasks including translation, summarization, and technical document analysis (Ming et al., 2022). Nevertheless, their application to ambiguity detection in software requirements engineering remains limited (Franke et al., 2024). Moreover, contextual embeddings alone may not adequately capture lexical vagueness specific to requirement documentation. Despite the effectiveness of transformer-based architectures, ambiguity detection in requirements engineering remains challenging due to class imbalance, implicit linguistic vagueness, and limited availability of annotated requirement corpora.

These observations indicate that existing approaches, whether rule-based, statistical, or purely contextual, remain insufficient when applied independently. There is therefore a need for a framework that integrates lexical, structural, and contextual representations to improve ambiguity-detection performance in requirements engineering.

Traditional ambiguity detection techniques, including rule-based systems and keyword-based searches, primarily identify surface-level issues and frequently generate high rates of false positives and false negatives due to their inability to capture semantic and contextual meaning (Ezzini et al., 2022). Classical machine learning classifiers such as Naïve Bayes, Logistic Regression, Support Vector Machines, and Decision Trees improved predictive performance using TF-IDF representations (Aqsa et al., 2021). However, TF-IDF captures only term frequency information and ignores word order, syntactic structure, and contextual relationships. Consequently, these models perform poorly when ambiguity arises from implicit intent, vague expressions, or domain-specific terminology, leading to low recall for ambiguous requirements (Liu et al., 2022).

Transformer-based models such as BERT, SBERT, and GPT address contextual representation through deep semantic modeling (Osman and Zaharin, 2018). Although SBERT generates sentence-level embeddings suitable for classification, when used independently it may overlook lexical ambiguity specific to requirement documents. Additionally, transformer-based approaches involve computational complexity and limited requirement-specific adaptation, restricting their practical deployment in requirements engineering.

Character-level n-gram features capture morphological and structural patterns (Sonbol et al., 2022), yet they do not encode sentence-level semantics. Moreover, classical classifiers present inherent limitations. Naïve Bayes assumes feature independence (Venkatesh and Ranjitha, 2018), Support Vector Machines are sensitive to class imbalance and sparse feature spaces (Evgeniou and Pontil, 2001), and tree-based models may favor majority classes when semantic signals are weak (Mehmood et al., 2023). These limitations indicate that single-feature approaches are insufficient for comprehensive ambiguity detection.

This study proposes a hybrid semantic–lexical ambiguity detection framework that integrates lexical, structural, and contextual representations within a unified learning architecture. Unlike conventional ambiguity detection approaches that rely on a single feature representation, the proposed methodology combines TF-IDF, character n-grams, and SBERT embeddings to capture complementary linguistic signals associated with ambiguous software requirements.

Unlike most existing ambiguity detection studies that evaluate lexical, structural, or contextual representations independently, this work investigates the complementary interaction of all three linguistic dimensions within a single classification framework. In addition to feature-level integration, the proposed methodology incorporates cross-dataset evaluation, stratified cross-validation, transformer-based comparative analysis, and statistical significance testing to assess robustness beyond dataset-specific optimization. Consequently, the contribution of this study extends beyond feature fusion by providing a systematically validated ambiguity detection framework that combines representation-level integration with rigorous empirical evaluation.

Furthermore, the proposed framework is evaluated across two heterogeneous requirements engineering datasets exhibiting different class imbalance characteristics. This cross-dataset evaluation provides evidence of robustness and generalizability beyond dataset-specific optimization. Existing ambiguity detection studies have only limitedly investigated the complementary interaction of lexical, structural, and contextual representations within a unified ambiguity classification framework combined with cross-validation-based robustness assessment and transformer-based comparative evaluation.

This study contributes to ambiguity detection in software requirements by introducing a hybrid semantic–lexical framework that integrates lexical, structural, and contextual representations within a unified learning architecture. The objectives of the study are:

- To evaluate the effectiveness of contextual sentence embeddings for ambiguity classification in software requirements.
- To compare semantic embeddings with traditional lexical and structural feature representations, including TF-IDF and character n-grams.
- The framework is evaluated across two heterogeneous requirements engineering datasets exhibiting different class imbalance characteristics, providing preliminary evidence of robustness across distinct dataset conditions.
- To evaluate model robustness using cross-dataset experimentation, stratified cross-validation, and transformer-based baseline comparison.

## 2 Background study

Software requirement ambiguity has long been recognized as a major challenge in requirements engineering. Considering that most software requirements can be represented in natural language, it is prone to misinterpretation, lexical ambiguity, and semantic ambiguity. This often results in delays, increased development cost, and in severe cases, project failure. Over time, research has evolved from rule-based approaches to machine learning and transformer-based models. More recently, transformer-based language models have enabled contextual interpretation of requirements by modeling semantic relationships between words and sentences.

### 2.1 Ambiguity as a requirements engineering concept

Although the ambiguity-detection task addressed in this study is operationalized as a binary text-classification problem, ambiguity itself is a well-established construct within Requirements Engineering (RE) theory rather than a purely linguistic or machine-learning phenomenon. The RE literature commonly distinguishes several forms of ambiguity: lexical ambiguity, where a single word admits multiple meanings; syntactic ambiguity, where a sentence structure permits more than one grammatical parse; semantic ambiguity, where the intended meaning of a proposition is underdetermined even when the syntax is unambiguous; pragmatic ambiguity, where the same statement can be interpreted differently depending on context or stakeholder intent; and referential ambiguity, where it is unclear which entity a pronoun or noun phrase denotes. A related but distinct concept is vagueness, in which a term admits borderline cases (for example, “fast” or “user-friendly”) without necessarily supporting multiple distinct readings; vague terms were treated as ambiguity indicators in this study because they introduce interpretive uncertainty of the kind targeted by requirements-quality frameworks, even though a strict linguistic taxonomy would separate vagueness from ambiguity proper. This taxonomy is broadly consistent with the quality characteristics defined for software requirements specifications in ISO/IEC/IEEE 29148, which identifies unambiguousness as one of the core attributes a well-formed requirement should exhibit, alongside completeness, consistency, verifiability, and traceability. Framed in these terms, the classifier proposed in this study operationalizes unambiguousness checking as a screening mechanism that can be embedded within requirements validation, requirements inspection, and requirements quality-assurance activities, flagging candidate statements for human review rather than replacing the analyst's judgement. This framing situates the contribution primarily within RE practice, with the machine-learning components serving as the mechanism through which an established RE quality attribute is operationalized at scale.

The initial methods of ambiguity detection were mainly rule-based. These systems were based on pre-defined linguistic rules, keyword identification or fixed pattern match. Veizaga et al. (2024) proposed a rule-based static analysis system capable of identifying and suggesting improvements for ambiguous natural language requirements. Although it was effective at detecting surface-level ambiguity, the technique was unsuccessful at detecting contextual meaning. Likewise, Gleich et al. (2010) came up with a text against-the-already-named tool-based solution, which elucidated the ambiguous textual requirement in terms of linguistic rules. It was not very strong in automation though it was useful in the training of the analysts and in the explanation of ambiguity. Lexical ambiguity refers to words that possess multiple meanings. The syntactic one, the ambiguous sentence structure, was put in linguistic taxonomies (Ezzini et al., 2022; Zhao et al., 2021). Osman and Zaharin (2018) also utilized pattern-based and keyword spotting rule-based tools. Such systems could recognize explicit ambiguity but they were weak in recognizing more complicated sentence forms and interpreting the surrounding context.

With the advancement of natural language processing (NLP), ambiguity detection shifted toward semantic-level analysis rather than purely lexical matching. Zhao et al. (2021) conducted a systematic mapping study and highlighted the role of semiotic NLP analysis in identifying inconsistency and vague structures in requirements. Sonbol et al. (2022) explored various NLP-based text representations to capture syntactic and semantic information, demonstrating that embedding-based models outperformed simple keyword-based detection. Mir Khatian et al. (2021) employed supervised machine learning classifiers with Bag-of-Words and TF-IDF features to classify non-functional requirements. Although promising, their model struggled to capture implicit or contextual ambiguity. Osman and Zaharin (2018) proposed a prototype combining text mining and multiple classifiers, achieving moderate performance on industrial datasets. However, most NLP-based approaches remained limited to lexical or syntactic levels and were tested on restricted datasets, reducing their generalizability (Rankovic, 2025).

Hybrid methods were subsequently proposed to combine machine learning methods with rule based reasoning. Osman and Zaharin (2018) performed TF-IDF feature extraction with sentiment analysis, clustering and Random Forest classifier to identify ambiguity in SRS documents, which they found to be highly accurate. Although the hybrid methods were more effective in detection than the single method approaches, they were also affected by quality of the data set and domain specificity. To generalize across requirement corpora, high accuracy according to controlled datasets did not always generalize (Ming et al., 2022).

The research on ambiguity detection was further developed with the help of cross-domain and semantic approaches. In the introduction of domain-specific terms with varying meanings in each professional situation, Liu et al. (2022) suggested determining the use of words that have varied meanings. Their approach was very accurate in fields like law, statistics, and biomedicine, but there has been noise in cross domain using it. Moharil and Sharma (Moharil and Sharma, 2022) used BERT based contextual embeddings to identify intra domain, inter-domain ambiguity in multi-domain SRS corpora with clustering. Their method showed the capacity to capture changes in context in the meaning of words, but it was highly dependent on large labeled datasets and cautious fine-tuning (Ezzini et al., 2021). These works have shown that semantic embeddings can bring about a latent ambiguity which is undiscovered by conventional methods of searching with key-words or syntactic.

Transformer-based architectures have contributed greatly to the ambiguity detection. Self-attention models like BERT and GPT do not process words in isolation, but rather with their contextual meaning, rather than analyzing the words (Alhamed and Storer, 2022). Moharil and Sharma (2022) demonstrated that the representations obtained through Bert were effective in identifying fine semantic variations in and across domains. In their study, Kaur and Kaur (2023) proposed a prompt-learning structure PRCBERT, a paradigm that enhances zero-shot classification accuracy. Yucalar (2023) introduced a BERT-BiCNN that combined transformer embeddings and CNN layers to facilitate structural feature extraction. In a systematic review that outlines 60 studies, Bahtiar et al. (2023) found that transformer-based models were always the best in the requirement classification tasks compared to the classical machine learning methods. Although these are the improvements, industrial adoption has not been high due to the cost of computation, scalability problems and integration challenges.

Methods of feature extraction have highly been developed as well. TF-IDF and n-gram are still popular approaches since they are simple and effective (Mir Khatian et al., 2021; Liu et al., 2022). Nevertheless, these lexical characteristics do not have an in-depth semantics and have problems with ambiguity in context. Distributed semantic representations, like skip-gram and GloVe word embeddings, generated better contextual representations (Sonbol et al., 2022). Most recently, contextual embeddings of transformer models including BERT and SBERT have been used in ambiguity detection (Moharil and Sharma, 2022). More specifically, SBERT produces sentence-level embeddings that are amenable to classification exercises (Reimers and Gurevych, 2019). Results of studies combining SBERT with traditional classifiers, including Logistic Regression and SVM, showed better recall and F1-scores than TF-IDF-based models, (Rankovic, 2025). However, SBERT can perhaps not suffice to represent lexical vagueness when ambiguous signs rely on specific requirement terms.

Baseline machine learning classifiers have been extensively used in requirements engineering. The Logistic Regression is also commonly used because it is simple and runs well with sparse TF-IDF features (Venkatesh and Ranjitha, 2018; Bahtiar et al., 2023). In high-dimensional spaces, Decision Trees are easily interpretable and susceptible to overfitting (Al-Azani and El-Alfy, 2017). Random Forest enhances generalization since several trees are combined, but interpretability deteriorates. Support Vector Machines (SVM) work well with text data of high dimension and sparseness, but can be affected by the problem of computational complexity and class imbalance. Naive Bayes is computationally efficient but based on existence of strong independence assumptions which are hardly true in natural language problems (Peretz et al., 2024). Although such models have established standard baseline requirements in detecting ambiguities, they cannot fully understand the contextual meaning as it is based on lexical features.

Recent studies have examined SVM in combination with contextual embeddings. Moharil and Sharma (Ferrar and Esuli, 2019) demonstrated that integrating SBERT embeddings with SVM significantly improved recall in detecting intra-domain and inter-domain ambiguity. A comprehensive evaluation combining lexical, structural, and contextual features within a unified ambiguity detection framework remains underexplored.

### 2.2 Limitations of existing ambiguity detection studies

Existing ambiguity detection approaches in requirements engineering continue to exhibit several important limitations. Many traditional studies rely heavily on single-feature representations such as TF-IDF, Bag-of-Words, or rule-based linguistic patterns, which primarily capture surface-level lexical information while failing to model deeper contextual ambiguity. Although transformer-based embeddings improve semantic understanding, they are frequently evaluated independently without effective integration of lexical and structural representations. In addition, many existing studies lack rigorous cross-dataset validation and are evaluated only on limited or domain-specific corpora, reducing their generalizability across diverse requirement environments. Comparative evaluation against modern transformer-based baselines also remains limited in several ambiguity detection frameworks. Furthermore, robustness evaluation is often insufficient, as many studies depend on a single train-test split without cross-validation or stability analysis. Class imbalance presents an additional challenge because ambiguous requirements are typically underrepresented, leading to low recall and unreliable minority-class detection. These limitations indicate the need for a more robust hybrid framework capable of integrating lexical, structural, and semantic representations within a unified ambiguity detection pipeline.

Despite substantial progress, several research gaps remain. Many classical models rely primarily on TF-IDF or Bag-of-Words features and fail to capture contextual ambiguity (Mir Khatian et al., 2021; Liu et al., 2022). Although transformer-based models demonstrate superior semantic understanding, their application to ambiguity detection remains limited and is often evaluated on small, domain-specific datasets (Ferrar and Esuli, 2019; Ming et al., 2022). Scalability, interpretability, and integration into real-world requirement engineering workflows remain open challenges (Juhnke et al., 2021; Franke et al., 2024). Most existing studies evaluate lexical, structural, or contextual representations independently. Limited research has systematically examined how complementary linguistic representations interact within a unified ambiguity modeling framework under cross-dataset evaluation settings.

Furthermore, existing literature does not provide a systematic hybrid framework that explicitly integrates TF-IDF, character n-grams, and SBERT embeddings for ambiguity classification. Most studies evaluate models in isolation rather than investigating the complementary strengths of lexical, structural, and contextual features. This lack of integrated feature fusion limits robustness and generalizability across diverse requirement datasets.

These gaps indicate the need for a hybrid semantic–lexical framework capable of integrating lexical, structural, and contextual representations within a unified ambiguity detection pipeline.

Table 1 presents a structured summary of the major approaches used in ambiguity detection within requirements engineering. The table highlights the progression from rule-based techniques to machine learning, hybrid, and transformer-based models. It also compares input representations, datasets, key findings, and limitations across studies. This comparison demonstrates that earlier methods relied on surface-level linguistic features, while recent approaches focus on contextual and semantic understanding. However, each category exhibits specific limitations, particularly in handling context and generalizability. Overall, the table supports the need for a hybrid semantic–lexical framework that integrates multiple feature representations for improved ambiguity detection.

**Table 1:** Background study on ambiguity detection in requirements engineering.

Category	References	Model/approach	Input representation	Dataset(s)	Key findings	Limitations
Rule-based methods	Veizaga et al. (2024)	Rule-based static analysis	Linguistic rules, patterns	SRS documents	Effective for detecting surface-level ambiguity	Cannot capture contextual meaning
	Gleich et al. (2010)	Tool-supported rule analysis	Textual rules, linguistic patterns	Requirement texts	Useful for training and explanation	Limited automation
	Osman and Zaharin (2018)	Pattern-based detection	Keywords, patterns	SRS datasets	Detects explicit ambiguity	Weak on complex sentence structures
NLP and ML methods	Zhao et al. (2021)	Semiotic NLP analysis	Linguistic + semantic features	Multiple studies	Identifies vague and inconsistent requirements	Limited depth in contextual understanding
	Sonbol et al. (2022)	Embedding-based NLP models	Word embeddings	Requirement datasets	Outperforms keyword-based methods	Requires pre-processing and tuning
	Mir Khatian et al. (2021)	Supervised ML classifiers	BoW, TF-IDF	Requirement corpora	Promising classification performance	Fails on implicit ambiguity
	Osman and Zaharin (2018)	Text mining + classifiers	TF-IDF, lexical features	Industrial datasets	Moderate performance	Limited generalizability
Hybrid approaches	Osman and Zaharin (2018)	TF-IDF + RF + clustering	Statistical + sentiment features	SRS documents	Improved accuracy over single models	Dataset dependency
	Ming et al. (2022)	Hybrid ML frameworks	Combined features	Controlled datasets	Better detection performance	Poor cross-domain generalization
Semantic and cross-domain models	Liu et al. (2022)	Domain-specific semantic detection	Contextual word usage	Multi-domain datasets	High accuracy in specialized domains	Noisy in cross-domain settings
	Moharil and Sharma (2022)	BERT-based clustering	Contextual embeddings	Multi-domain SRS	Captures semantic variation	Requires large labeled datasets
Transformer-based models	Alhamed and Storer (2022)	Transformer (BERT/GPT)	Contextual embeddings	Requirement datasets	Strong contextual understanding	High computational cost
	Kaur and Kaur (2023)	PRCBERT (prompt-based)	Contextual embeddings	Classification datasets	Improves zero-shot performance	Limited evaluation scope
	Yucalar (2023)	BERT + BiCNN	Hybrid embeddings + CNN features	Requirement datasets	Captures semantic + structural features	Complex architecture
	Bahtiar et al. (2023)	Systematic review (60 studies)	Various embeddings	Multiple datasets	Transformers outperform ML models	Scalability and integration issues
Feature engineering methods	Osman and Zaharin (2018)	TF-IDF, n-grams	Lexical features	Text corpora	Simple and effective baseline	Lacks semantic depth
	Sonbol et al. (2022)	Word embeddings (GloVe, skip-gram)	Distributed vectors	NLP datasets	Improved contextual representation	Limited sentence-level understanding
	Reimers and Gurevych (2019)	SBERT	Sentence embeddings	NLP datasets	Strong performance in classification	Limited lexical ambiguity handling
Baseline ML models	Venkatesh and Ranjitha (2018)	Logistic regression	TF-IDF	Text datasets	Efficient with sparse data	Limited semantic understanding
	Al-Azani and El-Alfy (2017)	Decision trees	Feature vectors	Text datasets	Interpretable models	Overfitting issues
	Peretz et al. (2024)	Random forest	Ensemble features	Requirement datasets	Improved generalization	Reduced interpretability
	Evgeniou and Pontil (2001)	SVM	High-dimensional vectors	Text datasets	Strong performance in text classification	Computational complexity
Hybrid with embeddings	Ferrar and Esuli (2019)	SBERT + SVM	Sentence embeddings	Requirement datasets	Improved recall and F1-score	Still lacks full feature integration

Direct numerical comparison with previously published ambiguity-detection studies should be interpreted cautiously because the reported studies differ substantially in datasets, ambiguity definitions, annotation procedures, class distributions, pre-processing pipelines, and evaluation protocols. For this reason, Table 2 provides a methodological comparison rather than ranking the proposed framework directly against previously reported performance values. The experimental comparison in this study is therefore based on models evaluated under the same dataset and experimental settings, including conventional lexical, structural, semantic, and hybrid configurations, together with a fine-tuned BERT baseline on the SRS dataset. This design allows internally controlled comparison while avoiding potentially misleading numerical comparisons across non-identical experimental settings.

**Table 2:** Comparison of the proposed framework with representative ambiguity detection approaches.

Approach	Representation	Dataset Scope	Model	Validation	Main Limitation/advantage
Osman and Zaharin (2018)	TF-IDF/lexical	SRS	ML classifiers	Dataset-based evaluation	Limited semantic representation
Ferrar and Esuli (2019)	NLP/semantic features	Requirements datasets	ML-based	Experimental evaluation	Does not integrate all three feature types
Moharil and Sharma (2022)	Transformer-based	Multi-domain SRS	Transformer	Experimental evaluation	Requires contextual modeling resources
Yucalar (2023)	BERT + BiCNN	Requirements dataset	Hybrid deep model	Empirical evaluation	More complex architecture
Proposed framework	TF-IDF + character n-grams + SBERT	SRS + FR-NFR	SVM and other classifiers	5-fold CV + BERT baseline + Wilcoxon test + Cohen's Kappa	Unified lexical, structural, and contextual representation evaluated across two datasets; broader generalizability requires further validation

It is worth clarifying the nature of the contribution relative to this existing hybrid-feature literature. Combining sparse lexical representations with dense contextual embeddings is not in itself a new idea in text classification, and several of the studies summarized in Table 1 already explore pairwise combinations of lexical and semantic features. The contribution of the present study is therefore best understood as empirical and architectural rather than purely algorithmic: to the authors' knowledge, it is the first study to integrate three complementary representations (TF-IDF, character n-grams, and SBERT) within a single ambiguity-detection pipeline and to examine that integration across two datasets with different characteristics through stratified cross-validation, an SRS-specific fine-tuned transformer baseline, Wilcoxon significance testing, and inter-annotator agreement analysis in combination.

The main contributions of this study are summarized as follows:

1. A hybrid semantic–lexical ambiguity detection framework is proposed that jointly integrates TF-IDF lexical features, character n-gram structural representations, and SBERT contextual embeddings within a unified learning architecture.
2. Unlike existing ambiguity detection studies that evaluate these representations independently, this work systematically evaluates the complementary interaction of lexical, structural, and contextual representations for ambiguity classification.
3. The framework is evaluated across two heterogeneous requirements engineering datasets exhibiting different linguistic characteristics and class imbalance profiles, providing preliminary evidence of robustness beyond a single dataset.
4. The framework is evaluated using stratified five-fold cross-validation, an SRS-specific transformer baseline comparison, and exploratory statistical analysis using the Wilcoxon signed-rank test.
5. Annotation reliability is assessed through inter-annotator agreement analysis using Cohen's Kappa coefficient.

## 3 Research methodology

This study proposes a hybrid semantic–lexical framework for detecting ambiguity in software requirement statements using a hybrid natural language processing approach. The framework integrates lexical, structural, and semantic feature representations in order to capture multiple linguistic characteristics that contribute to ambiguity in requirements documentation. The methodology follows a structured pipeline beginning with dataset preparation and text pre-processing, followed by feature extraction, hybrid feature integration, and supervised classification. The overall objective is to construct a unified representation capable of identifying ambiguous requirements more effectively than traditional single-feature approaches.

Figure 1 illustrates the overall architecture of the proposed ambiguity detection framework. Requirement statements are first pre-processed and transformed into structured representations. Three complementary feature types are then extracted, including TF–IDF lexical features, character n-gram structural features, and contextual semantic embeddings generated using Sentence-BERT. These features are subsequently combined into a hybrid representation that serves as input to supervised classifiers. Finally, the trained models are evaluated using standard classification metrics. All experiments were conducted using Python 3.11. Random seeds were fixed at 42 for data splitting, SMOTE oversampling, and classifier training to ensure experimental reproducibility. Identical pre-processing, feature extraction, and evaluation procedures were applied across all datasets.

**Figure 1:**
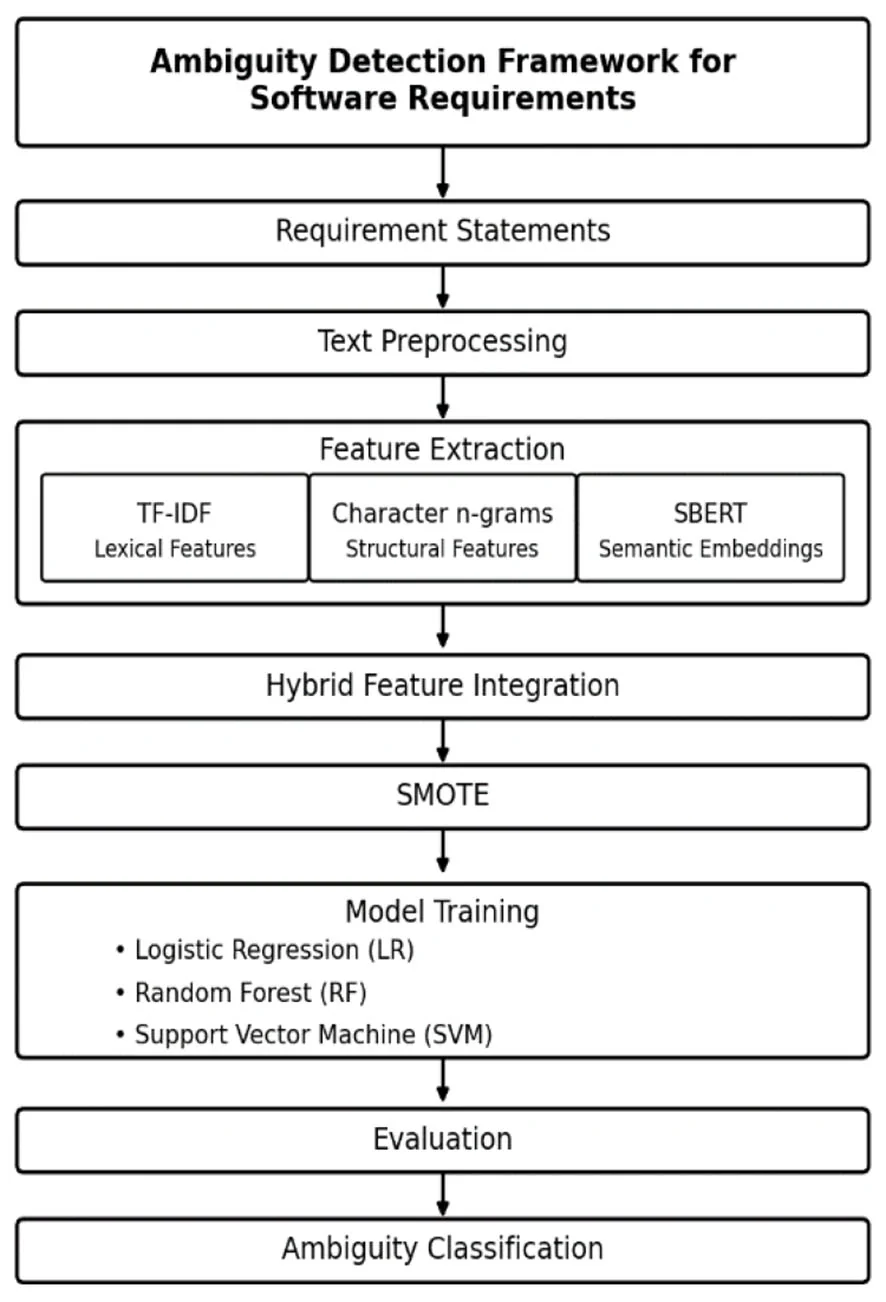
Proposed hybrid ambiguity detection framework.

### 3.1 Dataset preparation

Two publicly available datasets were used to evaluate the effectiveness of the proposed framework.

The first dataset, obtained from Vaibhav (2023), consists of 976 software requirement statements derived from a publicly available Software Requirements Specification (SRS) corpus. The original dataset contains four requirement categories, namely Functional Requirements, Non-Functional Requirements, Usability Requirements, and Operational Requirements. Since the objective of this study is ambiguity detection rather than requirement-type classification, the dataset was reformulated into a binary ambiguity classification task.

The second dataset (Sonali, 2024) contains 6,008 requirement statements collected from functional and non-functional requirement repositories in software engineering projects. After pre-processing, duplicate removal, and data standardization, this dataset was similarly transformed into a binary ambiguity classification setting to ensure methodological consistency across all experiments.

For both datasets, requirement statements were categorized into either clear (0) or ambiguous (1) classes using ambiguity identification guidelines derived from established requirements engineering literature. The labeling protocol considered vague expressions, underspecified intent, contextual uncertainty, optional interpretations, and non-measurable quality descriptions as potential indicators of ambiguity. Ambiguity-related cue indicators were employed only as auxiliary consistency-supporting signals during dataset preparation and were not used to directly assign class labels. Final ambiguity verification was performed through manual review using the defined annotation criteria to minimize heuristic bias and improve labeling reliability.

Each requirement instance is represented as a pair *(s*_*i*_*, y*_*i*_*)*, where *s*_*i*_ denotes the requirement statement and *y*_*i*_ ε *{0,1}* denotes its ambiguity label. Reformulating both datasets into a unified binary classification setting enables direct evaluation of ambiguity detection performance while maintaining experimental consistency across heterogeneous requirement sources.

To assess annotation reliability, a randomly selected subset of 200 requirement statements was independently reviewed using the ambiguity classification criteria adopted in this study. Inter-annotator agreement was measured using Cohen's Kappa coefficient. The obtained agreement score of **κ** **=** **0.80** indicated substantial agreement between annotation decisions, demonstrating that ambiguity labels were assigned consistently and were not excessively dependent on subjective judgement. Any disagreements were subsequently reviewed and resolved to ensure alignment with the ambiguity identification guidelines used throughout dataset preparation.

The annotation was carried out by the first author, who has post-graduate training in software requirements engineering and applied the pre-defined labeling guidelines under the supervision of the co-authors. The annotation process involved review of the ambiguity taxonomy, examples of vague and context-dependent requirements, and discussion of borderline cases before the final labeling stage. A second reviewer independently assessed a randomly selected subset of 200 statements to evaluate annotation consistency. The disagreements identified during the reliability check were resolved through discussion against the documented ambiguity criteria rather than by majority vote, since only a single primary annotator assigned the working labels. Additional external annotators with varying levels of industrial experience were not employed, which is acknowledged as a limitation of the current annotation design.

### 3.2 Text pre-processing

All requirement statements were normalized before feature extraction. The pre-processing pipeline included lowercasing, removal of punctuation and special characters, tokenization, stopwords removal using the NLTK corpus, and lemmatization. These steps reduce lexical noise while preserving semantic content.

To support ambiguity consistency analysis, a cue-word mechanism was introduced using ambiguity-related expressions such as “may,” “might,” “could,” “possibly,” “fast,” “quickly,” “easy,” “usually,” “often,” and “user-friendly”. The resulting binary cue indicators were employed only as auxiliary linguistic signals during pre-processing and analysis and were not used to directly assign ambiguity labels. Aggressive pre-processing was applied for TF-IDF and character n-gram extraction, whereas minimally altered sentences were retained for SBERT encoding to preserve contextual semantics.

### 3.3 Feature representation

Ambiguity in software requirements may arise from lexical vagueness, structural phrasing, or contextual meaning. To capture these different aspects, three complementary feature representations were extracted.

#### 3.3.1 TF-IDF representation

Term Frequency–Inverse Document Frequency (TF–IDF) is widely used to represent lexical importance within textual documents. The term frequency component measures how frequently a word appears in a given sentence, while the inverse document frequency component reduces the influence of frequently occurring terms across the entire dataset. Term frequency is defined as:


$$\begin{array}{l} TF\;\left(t,\;d\right)\;=\frac{count\;of\;\left(t,\;d\right)}{\left|d\right|} \end{array}$$

Where count(t,d) represents the number of occurrences of term t in document d and |d| denotes the total number of terms in the document.

Inverse document frequency is calculated as:


$$\begin{array}{l} IDF\left(t\right)\;=\;log\;\left(\frac{N}{\;1+df\;\left(t\right)}\right) \end{array}$$

Where N represents the total number of documents and df (t) denotes the number of documents containing term t.

The final weight is computed as:


$$\begin{array}{l} TF-IDF\left(t,d\right)=TF\left(t,d\right)\times IDF\left(t\right) \end{array}$$

Unigram, bigram, and trigram features were extracted to capture lexical patterns that may indicate ambiguity.

**Example:**

For the sentence:

“The system shall respond quickly under all conditions”.

If the term “respond” appears once in a 5-word sentence, then

TF = 1/5 = 0.2

Assuming df = 50 and N=1,000:

IDF ≈ log(1,000/51) ≈ 2.99

TF-IDF ≈ 0.2 × 2.99 ≈ 0.598

#### 3.3.2 Character n-gram representation

Character n-gram features capture sub-word structural patterns and morphological variations within requirement statements. These features are particularly useful for identifying vague expressions or structural patterns that may not be visible at the word level.

Character n-grams of length three to five were extracted from each requirement sentence. For example, the word *respond* generates character trigrams such as *res, esp, spo, pon*, and *ond*. Similarly, the word *quickly* generates trigrams such as *qui, uic, ick, ckl*, and *kly*.

These structural representations enable the model to recognize ambiguity cues embedded within word structures and phrasing patterns.

#### 3.3.3 SBERT embeddings

Sentence-BERT (SBERT) was used to generate contextual sentence embeddings that capture semantic meaning. SBERT extends the BERT architecture by producing fixed-length vector representations suitable for semantic similarity and classification tasks.

Each requirement sentence was encoded into a dense semantic vector of dimension 384 using a pre-trained SBERT model. These embeddings capture contextual meaning by considering relationships between words within the sentence.

For example, the sentences:

“The system must respond very quickly.”

“The system shall be fast in responding.”

Contain different lexical expressions but convey similar semantic meaning. SBERT embeddings enable the model to capture such contextual similarity. Unlike end-to-end transformer fine-tuning, SBERT was employed as a semantic embedding mechanism to provide contextual sentence representations within the hybrid feature space.

### 3.4 Hybrid feature integration

Lexical, structural, and semantic features capture different aspects of ambiguity in natural language requirements. To exploit the complementary strengths of these representations, the extracted features were combined into a unified hybrid feature space. Let a requirement sentence be represented as s. Three feature mappings were obtained from the feature extraction stage:


$$\begin{array}{l} xtfidf\left(s\right)\in {\mathbb{R}}^{m}\\ xchar\left(s\right)\;\in {\mathbb{R}}^{k}\\ xsbert\left(s\right)\in {\mathbb{R}}^{384} \end{array}$$

Where x_tfidf_(s) denotes the lexical TF–IDF representation, x_char_(s) represents structural character n-gram features with dimensionality k = 3,000 (matching the max_features setting reported in Table 2), and x_sbert_(s) corresponds to the contextual semantic embedding generated by Sentence-BERT.

To ensure balanced contribution from each feature type, the feature vectors were first normalized using L2 normalization. The final hybrid representation was then constructed through vector concatenation:


$$\begin{array}{l} xhybrid\left(s\right)=\hat{x}tfidf\;⊕\;\hat{x}char\;⊕\;\hat{x}sbert \end{array}$$

Where ⊕ denotes vector concatenation. This unified representation simultaneously captures lexical importance, structural patterns, and contextual semantics, enabling the model to detect ambiguity more effectively.

The proposed integration strategy combines complementary linguistic representations within a unified learning space to model ambiguity across lexical, structural, and semantic dimensions. Unlike conventional feature engineering approaches that utilize lexical, structural, or semantic representations independently, the proposed integration strategy explicitly models ambiguity across multiple linguistic dimensions simultaneously. This design enables the framework to capture explicit lexical ambiguity cues, structural uncertainty patterns, and implicit contextual ambiguity within a single representation, thereby improving ambiguity detection robustness across heterogeneous requirements datasets.

Furthermore, the proposed framework treats ambiguity as a multi-dimensional linguistic phenomenon rather than a purely lexical classification problem. This distinction differentiates the proposed methodology from conventional ambiguity detection approaches that rely on isolated feature representations. Algorithm 1 of hybrid ambiguity detection framework is given as;

Algorithm 1 Hybrid semantic-lexical framework for ambiguity detection in software requirements.

 Require: Requirement sentences (S); TF–IDF lexical features; Character n-gram structural features; SBERT semantic embeddings; labeled training data
 Ensure Predicted ambiguity label y **∈** {0,1} for each requirement statement
1:  Collect requirement statements from the SRS dataset and the FR–NFR dataset.
2:  Apply text pre-processing including lowercasing, punctuation removal, tokenisation, stopword removal, and lemmatisation.
3:  Generate TF–IDF lexical feature vectors for each requirement statement.
4:  Extract character n-gram structural features (n = 3–5) from the requirement text.
5:  Generate contextual semantic embeddings using the pre-trained SBERT model.
6:  Apply L2 normalisation to TF–IDF, character n-gram, and SBERT feature vectors.
7:  Construct the hybrid feature representation by concatenating the three feature vectors.
8:  Apply SMOTE to the training data to address class imbalance in ambiguous requirement samples.
9:  Train supervised classifiers including Logistic Regression, Random Forest, and Support Vector Machine using the hybrid feature vectors.
10:  Predict the ambiguity class for each requirement statement using the trained classifier.
11:  Assign the predicted ambiguity label y ε {0,1}.
12:  Evaluate model performance using accuracy, precision, recall, and F1-score.

### 3.5 Handling class imbalance

Both datasets contain a significantly smaller proportion of ambiguous requirements. To mitigate majority-class bias, Synthetic Minority Oversampling Technique (SMOTE) was applied to hybrid training sets. To prevent information leakage, SMOTE was applied exclusively to training partitions after train-test splitting. During cross-validation, oversampling was performed independently within each training fold.

Synthetic samples are generated as:


$$\begin{array}{l} Xnew=xi+\lambda \left(xnn-xi\right) \end{array}$$

Where xi is a minority instance, xnn is its nearest neighbor, and λ ε (0,1). SMOTE was applied only to the training partitions used by the evaluated machine-learning classifiers. Test partitions remained unchanged to ensure unbiased performance evaluation.

### 3.6 Model training

The hybrid feature vectors were used to train supervised classifiers for ambiguity detection. Three widely used machine learning algorithms were selected, including Logistic Regression (LR), Random Forest (RF), and Support Vector Machine (SVM). These classifiers were chosen due to their effectiveness in high-dimensional textual classification tasks and their widespread use in natural language processing applications.

Since the datasets contain a relatively smaller number of ambiguous requirement statements, the Synthetic Minority Oversampling Technique (SMOTE) was applied to the training data to address class imbalance and improve minority-class representation. The classifiers were trained using the hybrid feature vectors obtained from TF–IDF lexical features, character n-gram structural features, and SBERT semantic embeddings. In addition to classical machine learning classifiers, a fine-tuned BERT-base model was evaluated as a transformer-based baseline for comparative analysis.

To assess whether the observed performance differences between the proposed Hybrid SVM model and the TF-IDF SVM baseline were statistically meaningful, a Wilcoxon signed-rank test was applied to the fold-level F1-scores from five stratified cross-validation folds. The comparison used the same SVM classifier to isolate the effect of feature representation. Because only five paired folds were available, the Wilcoxon analysis was treated as an exploratory statistical assessment rather than definitive evidence of population-level superiority. Statistical significance was evaluated at α = 0.05.

### 3.7 Evaluation metrics

The datasets were divided using an 80:20 stratified split to preserve class distribution. Experiments were initially evaluated using a stratified 80:20 train-test split. To improve robustness and reduce split-specific bias, additional 5-fold stratified cross-validation experiments were conducted. Mean and standard deviation across folds were reported for recall, F1-score, and accuracy. Model performance was evaluated using accuracy, precision, recall, F1-score, balanced accuracy, and Matthews correlation coefficient (MCC). Accuracy provides an overall measure of correct classification, whereas precision, recall, and F1-score focus on minority-class detection. Balanced accuracy accounts for performance across both classes, while MCC provides a correlation-based measure that remains informative under class imbalance


$$\begin{array}{l} Accuracy=\;\frac{TP+TN}{TP+TN+FP+FN}\\ Precision=\;\frac{TP}{TP+FP}\\ Recall=\;\frac{TP}{TP+FN}\\ F1-score=\;\frac{2^{*}Recall^{*}precision}{Recall+precision} \end{array}$$

For comparative ranking in Tables 3, 4, an Overall Score was additionally calculated as the arithmetic mean of accuracy, precision, recall, and F1-score:


$$\begin{array}{l} Overall\;Score=\frac{Accuracy+Precision+Recall+F1-score}{4} \end{array}$$

**Table 3:** Experimental configuration of pre-processing.

Component	Configuration
TF-IDF	ngram_range=(1,3), max_features=3000
Character N-Gram	analyzer=‘char', ngram_range=(3,5), max_features=3000
SBERT	all-MiniLM-L6-v2
SVM	LinearSVC(C=1.0, max_iter=5000)
Logistic regression	max_iter=1000
Random forest	n_estimators=100
SMOTE	random_state=42
Cross-validation	Stratified 5-fold
Random seed	42

**Table 4:** Cross-validation performance on SRS dataset.

Feature representation	Model	Mean F1-score	F1 Std Dev	Mean recall	Recall Std Dev	Mean accuracy	Accuracy Std Dev	Observation
TF-IDF + Char N-Gram + SBERT	LR	0.6775	0.0458	0.6194	0.0719	0.9237	0.0114	Most stable hybrid configuration
TF-IDF + Char N-Gram + SBERT	SVM	0.6696	0.0508	0.5868	0.0765	0.9257	0.0069	Strong hybrid generalization
TF-IDF	SVM	0.6123	0.0801	0.6098	0.1021	0.9009	0.0168	Stable lexical baseline
TF-IDF + Char N-Gram	SVM	0.6112	0.0875	0.5938	0.1024	0.9030	0.0188	Improved structural sensitivity
TF-IDF + Char N-Gram	LR	0.5940	0.0755	0.5154	0.0619	0.9082	0.0182	Moderate hybrid stability
TF-IDF	LR	0.5671	0.0507	0.4677	0.0386	0.9071	0.0109	High precision but lower recall
TF-IDF + Char N-Gram	RF	0.4729	0.1051	0.3822	0.1116	0.8927	0.0147	Recall affected by imbalance
Char N-Gram	SVM	0.4284	0.0405	0.4526	0.0426	0.8421	0.0201	Structural features alone insufficient
Char N-Gram	RF	0.4219	0.1155	0.3582	0.1332	0.8782	0.0148	High fold variability
TF-IDF	RF	0.4125	0.0500	0.2702	0.0480	0.9009	0.0051	Strong accuracy but weak ambiguity recall
Char N-Gram	LR	0.3913	0.0496	0.3655	0.0660	0.8534	0.0091	Limited contextual understanding
TF-IDF + Char N-Gram + SBERT	RF	0.3592	0.1103	0.2372	0.0877	0.8937	0.0113	Hybrid representation underutilized by RF

This composite value was used only as a descriptive ranking indicator to facilitate comparison among model configurations and was not treated as a standard performance metric or as evidence of statistical superiority. Because ambiguity detection involves class imbalance, model interpretation remains primarily based on precision, recall, F1-score, balanced accuracy, MCC, and the cross-validation analysis.

## 4 Results and analysis

This section presents the experimental evaluation of the proposed hybrid ambiguity detection framework using two benchmark datasets. The results are analyzed to examine the impact of different feature representations and classification models on ambiguity detection performance. The analysis first presents results for each dataset separately and then provides a comparative discussion of feature representations and classifiers.

Model performance is evaluated using accuracy, precision, recall, and F1-score. Since ambiguity detection involves class imbalance, recall and F1-score are particularly important for evaluating the ability of models to correctly identify ambiguous requirement statements.

In addition to standard train-test evaluation, stratified 5-fold cross-validation experiments were conducted to evaluate model robustness and reduce split-specific bias. Transformer-based baseline comparison using fine-tuned BERT was also performed to assess the effectiveness of the proposed hybrid semantic-lexical framework against modern contextual learning approaches.

### 4.1 Results on SRS dataset

The first experiments were done on the Software Requirements dataset that comprised a total of 976 requirements. They identified 747 clear (non-ambiguous) and 229 ambiguous indicating a moderate imbalance in classes. The dataset exhibits moderate class imbalance, which influences recall and F1-score. Table 5 presents the performance comparison of different feature representations and classification models on the SRS dataset.

**Table 5:** Software requirements dataset performance comparison.

Feature set	Model	Accuracy	Precision	Recall	F1-score	Overall score	Highlight
TF-IDF	SVM	0.909091	0.879518	0.708738	0.784946	0.820573	
TF-IDF	LR	0.856818	0.976190	0.398058	0.565517	0.699146	
TF-IDF	RF	0.877273	1.000000	0.475728	0.644737	0.749435	
CHAR N-GRAM	SVM	0.843182	0.723684	0.533981	0.614525	0.678843	
CHAR N-GRAM	LR	0.829545	0.818182	0.349515	0.489796	0.621760	
CHAR N-GRAM	RF	0.838636	0.758065	0.456311	0.569697	0.655677	
SBERT	SVM	0.868182	0.777778	0.611650	0.684783	0.735598	
SBERT	LR	0.822727	0.820513	0.310680	0.450704	0.601156	
SBERT	RF	0.809091	1.000000	0.184466	0.311475	0.576258	
TF-IDF+CHAR N-GRAM	SVM	0.897727	0.829545	0.708738	0.764398	0.800102	
TF-IDF+CHAR N-GRAM	LR	0.859091	0.836066	0.495146	0.621951	0.703064	
TF-IDF+CHAR N-GRAM	RF	0.843182	0.774194	0.466019	0.581818	0.666303	
TF-IDF+SBERT	SVM	0.906818	0.844444	0.737864	0.787565	0.819173	
TF-IDF+SBERT	LR	0.872727	0.898305	0.514563	0.654321	0.734979	
TF-IDF+SBERT	RF	0.822727	1.000000	0.242718	0.390625	0.614017	
CHAR N-GRAM+SBERT	SVM	0.879545	0.765957	0.699029	0.730964	0.768874	
CHAR N-GRAM+SBERT	LR	0.847727	0.800000	0.466019	0.588957	0.675676	
CHAR N-GRAM+SBERT	RF	0.838636	0.833333	0.388350	0.529801	0.647530	
TF-IDF+CHAR N-GRAM+SBERT	SVM	0.911364	0.833333	0.776699	0.804020	0.831354	BEST HYBRID (Overall Score)
TF-IDF+CHAR N-GRAM+SBERT	LR	0.879545	0.867647	0.572816	0.690058	0.752517	HYBRID
TF-IDF+CHAR N-GRAM+SBERT	RF	0.854545	0.914894	0.417476	0.573333	0.690062	HYBRID

The results demonstrate clear performance differences across feature representations. Models trained using single feature types such as TF-IDF, character n-grams, or SBERT embeddings show moderate performance, but their ability to detect ambiguous requirements remains limited. In particular, Logistic Regression shows high precision but relatively low recall when trained with TF-IDF features, indicating that many ambiguous requirements are incorrectly classified as clear.

Structural character n-gram features improve recall slightly compared with lexical features, as they capture structural patterns and vague expressions within requirement sentences. However, these features alone are not sufficient to consistently detect contextual ambiguity.

Semantic representations based on SBERT embeddings provide improved contextual understanding compared with purely lexical features. Nevertheless, SBERT-only models still exhibit unstable precision–recall behavior under class imbalance.

Significant improvements are observed when feature representations are combined. Pairwise combinations such as TF-IDF + SBERT and TF-IDF + character n-grams show clear improvements in recall and F1-score compared with single-feature models.

The strongest performance is achieved using the full hybrid representation that combines TF-IDF, character n-grams, and SBERT embeddings. When evaluated using Support Vector Machine, the hybrid model achieves the highest F1-score (0.8040) and the most balanced precision–recall trade-off. This result indicates that ambiguity detection benefits from integrating lexical, structural, and semantic information within a unified representation.

As illustrated in Figure 2, hybrid feature configurations consistently outperform single-feature models in terms of recall and F1-score. While Figure 3 highlights the performance of the hybrid SVM model on the SRS dataset.

**Figure 2:**
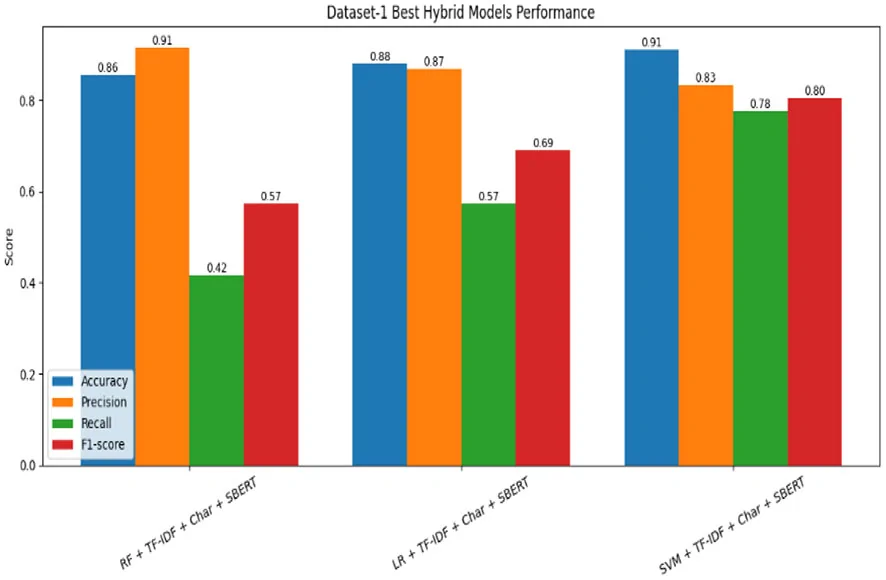
Performance comparison of single-feature and hybrid representations on the SRS dataset, showing the improvement in recall and F1-score achieved through feature integration.

**Figure 3:**
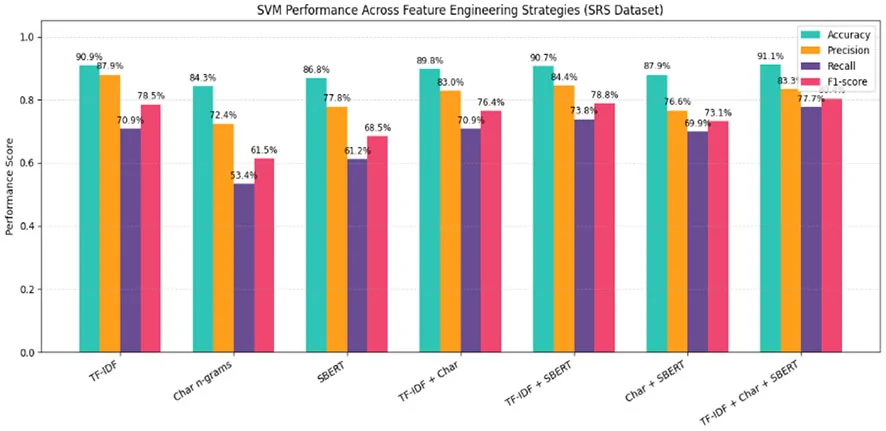
Performance of the hybrid SVM model on the SRS dataset, demonstrating the most balanced precision–recall trade-off among the evaluated configurations.

### 4.2 Cross-validation analysis on SRS dataset

To evaluate model stability and robustness, additional 5-fold stratified cross-validation experiments were performed on the SRS dataset using the strongest-performing configurations. Results demonstrate that the hybrid SVM model maintained stable recall and F1-score across folds with low standard deviation, indicating consistent ambiguity detection performance independent of dataset partitioning. In comparison, single-feature models exhibited greater variability, particularly under minority-class imbalance conditions.

The cross-validation results demonstrate that hybrid feature integration improves model robustness and stability across multiple folds. The hybrid configurations consistently achieved higher mean F1-scores and recall values compared with single-feature models, confirming the complementary contribution of lexical, structural, and semantic representations. In particular, the hybrid Logistic Regression and hybrid Support Vector Machine models maintained stable performance with relatively low standard deviation values, indicating reduced sensitivity to dataset partitioning. Figure 4 illustrates the mean F1-scores and corresponding standard deviations of the hybrid LR, SVM, and RF configurations across the five folds. In contrast, single-feature and Random Forest configurations exhibited larger variability and weaker minority-class recall under class imbalance conditions.

**Figure 4:**
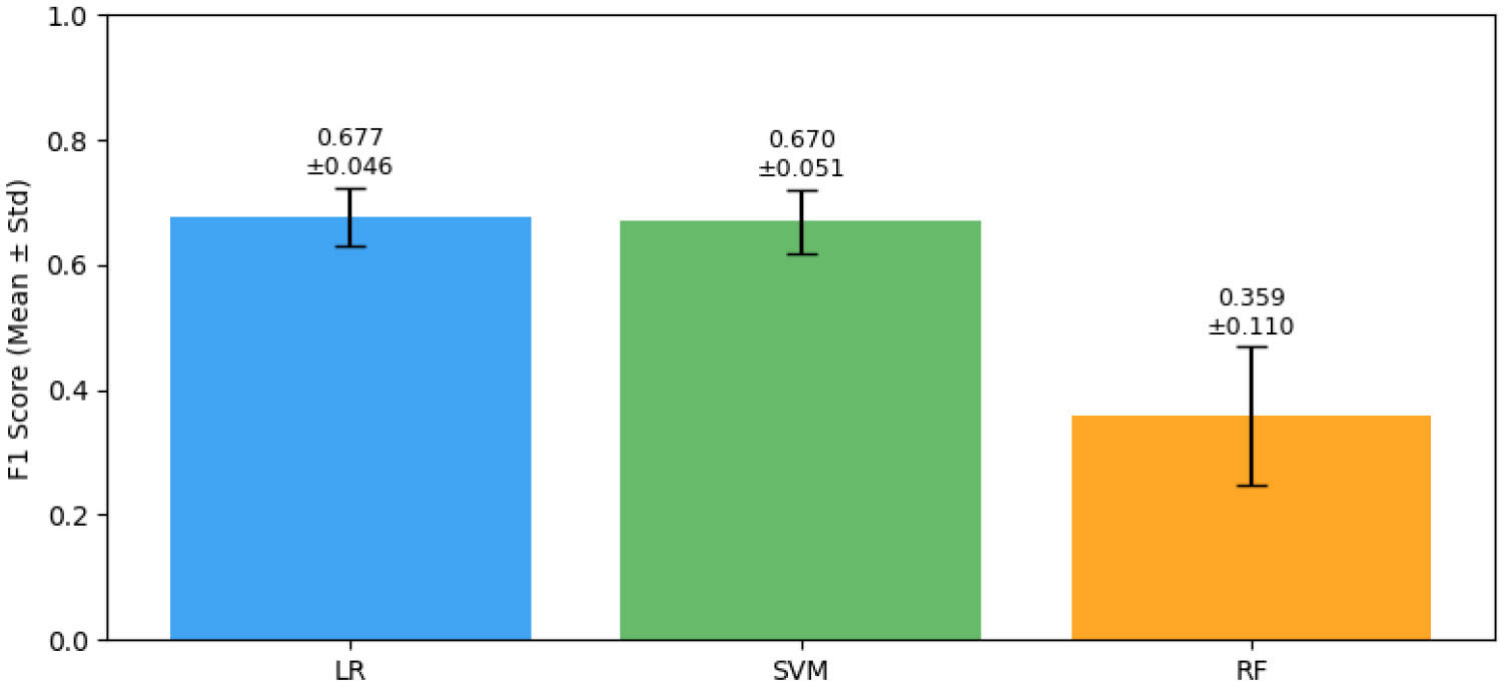
Mean F1-score and standard deviation across five-fold cross-validation for hybrid configurations on the SRS dataset, illustrating the stability of the proposed hybrid representation across data partitions.

### 4.3 Results on the FR–NFR dataset

The second set of experiments was conducted on the Functional–Non-Functional Requirements (FR-NFR) dataset, which contains 6,008 requirement statements. After pre-processing, 5,665 requirements were labeled as clear and 343 as ambiguous, indicating a greater class imbalance compared with the SRS dataset. Table 6 presents the classification performance across feature configurations and models on the FR-NFR dataset.

**Table 6:** Comparison table of FR-NFR.

Feature set	Model	Accuracy	Precision	Recall	F1-score	Overall score	Highlight
TF-IDF	SVM	0.717086	0.120497	0.629870	0.202294	0.417437	
TF-IDF	LR	0.760355	0.133531	0.584416	0.217391	0.423923	
TF-IDF	RF	0.925666	0.374332	0.454545	0.410557	0.541275	
CHAR N-GRAM	SVM	0.874300	0.179300	0.337700	0.234200	0.406375	
CHAR N-GRAM	LR	0.881700	0.192600	0.337700	0.245300	0.414325	
CHAR N-GRAM	RF	0.926400	0.200000	0.097400	0.131000	0.338700	
SBERT	SVM	0.934500	0.430300	0.461000	0.445100	0.567725	
SBERT	LR	0.932700	0.422200	0.493500	0.455100	0.575875	
SBERT	RF	0.953800	0.837200	0.233800	0.365500	0.597575	
TF-IDF + CHAR N-GRAM	SVM	0.965607	0.722628	0.642857	0.680412	0.752876	
TF-IDF + CHAR N-GRAM	LR	0.965976	0.750000	0.603896	0.669065	0.747234	
TF-IDF + CHAR N-GRAM	RF	0.957840	0.934783	0.279221	0.430000	0.650461	
TF-IDF + SBERT	SVM	0.970400	0.798400	0.642900	0.712200	0.780975	
TF-IDF + SBERT	LR	0.968600	0.800000	0.597400	0.684000	0.762500	
TF-IDF + SBERT	RF	0.954900	0.833300	0.259700	0.396000	0.610975	
CHAR N-GRAM + SBERT	SVM	0.934500	0.430300	0.461000	0.445100	0.567725	
CHAR N-GRAM + SBERT	LR	0.932700	0.422200	0.493500	0.455100	0.575875	
CHAR N-GRAM + SBERT	RF	0.953800	0.837200	0.233800	0.365500	0.597575	
TF-IDF + CHAR N-GRAM + SBERT	SVM	0.973743	0.854701	0.649351	0.738007	0.803951	BEST HYBRID
TF-IDF + CHAR N-GRAM + SBERT	LR	0.966716	0.750000	0.623377	0.680851	0.755236	HYBRID
TF-IDF + CHAR N-GRAM + SBERT	RF	0.955251	0.945946	0.227273	0.366492	0.623740	HYBRID

Similar performance patterns are observed in this dataset. Single-feature models demonstrate limited ability to detect ambiguous requirements, particularly under severe class imbalance. For example, TF-IDF-based models achieve high accuracy but extremely low precision for ambiguous instances, indicating that accuracy alone is not a reliable indicator of performance in this task.

Semantic SBERT embeddings improve contextual modeling but still produce unstable recall when used independently. Structural character n-gram features capture morphological patterns but fail to provide consistent ambiguity detection when used alone.

As observed in the SRS dataset, hybrid feature representations produce the most reliable results. The combination of TF-IDF, character n-grams, and SBERT embeddings consistently improves recall and F1-score across all classifiers.

The best overall performance is achieved by Support Vector Machine using the full hybrid feature representation, with an accuracy of 0.9737 and an F1-score of 0.7380. These results indicate that hybrid feature integration improves the model's ability to detect ambiguous requirements even in highly imbalanced datasets.

Figures 5, 6 illustrate the performance comparison across models and highlight the effectiveness of the hybrid SVM configuration.

**Figure 5:**
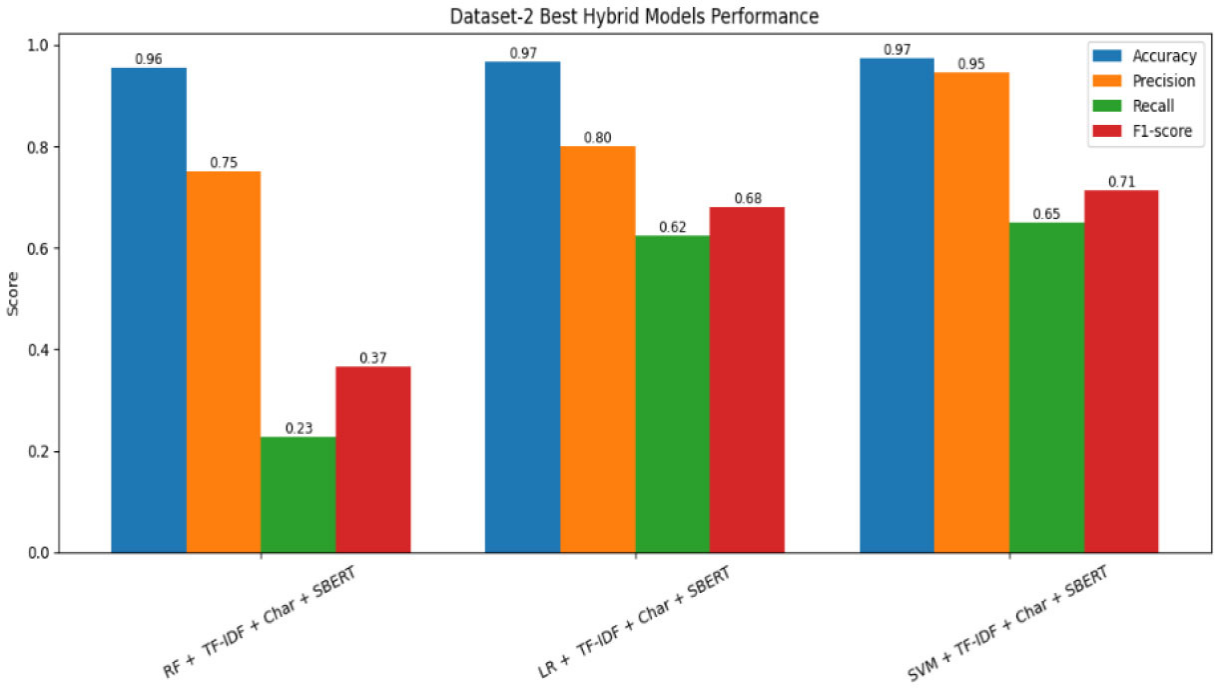
Performance comparison of hybrid feature configurations on the FR-NFR dataset, showing the improved recall–F1 trade-off achieved by the full TF-IDF, character n-gram, and SBERT representation.

**Figure 6:**
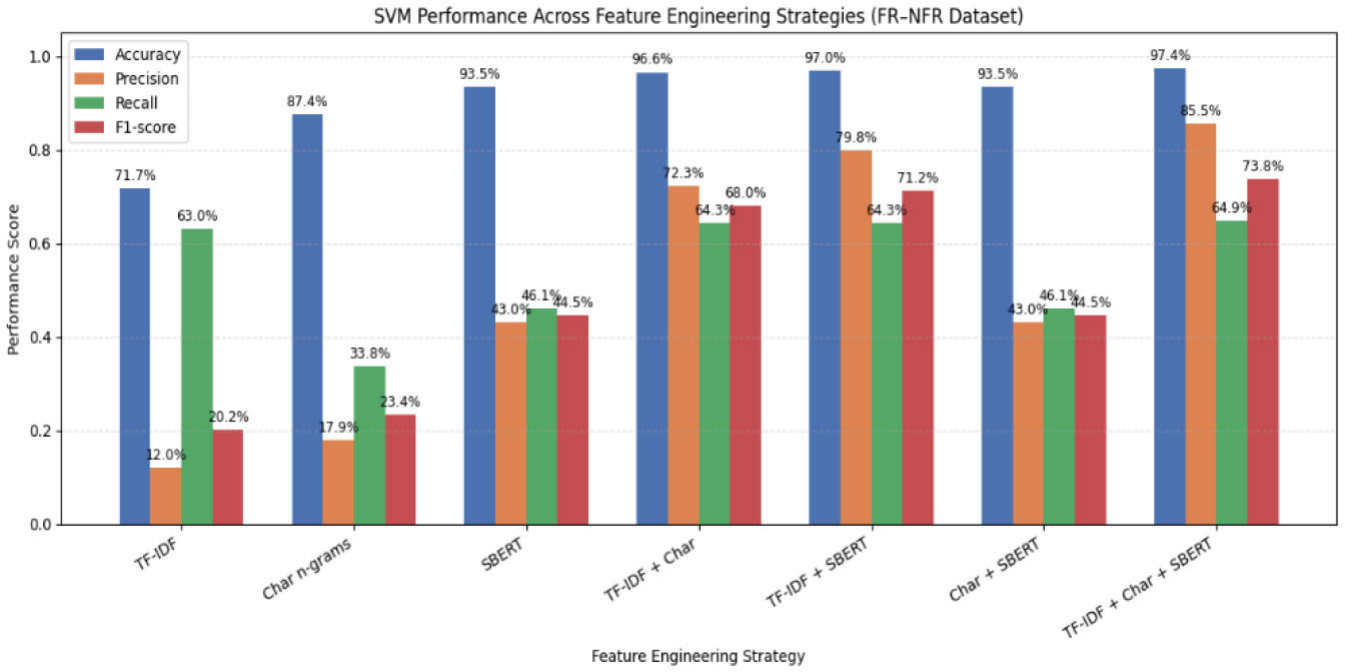
Performance of the hybrid SVM model on the FR-NFR dataset, showing its strongest F1-score and balanced performance under severe class imbalance.

### 4.4 Transformer baseline comparison

To further evaluate contextual learning capability, transformer-based baseline experiments were conducted using fine-tuned BERT. The transformer baseline demonstrated strong semantic modeling performance; however, the proposed hybrid representation achieved a more balanced precision–recall trade-off under severe class imbalance conditions. These findings suggest that combining lexical, structural, and semantic representations improves ambiguity detection stability across heterogeneous requirement datasets. The transformer baseline comparison is presented in Table 7.

**Table 7:** Transformer baseline comparison on SRS dataset.

Model	Accuracy	Precision	Recall	F1-score	Observation
BERT-base (fine-tuned)	0.9536	0.9444	0.6800	0.7907	Strong contextual semantic modeling
TF-IDF + Char N-Gram + SBERT + SVM	0.9114	0.8333	0.7767	0.8040	Best balanced hybrid performance

The transformer-based BERT model demonstrated strong contextual semantic learning capability and achieved the highest overall accuracy on the SRS dataset. The proposed hybrid framework achieved a marginally higher F1-score and recall than the fine-tuned BERT baseline. While the performance difference is relatively small, the results indicate that integrating lexical, structural, and semantic representations can provide competitive ambiguity detection performance. The hybrid approach may also offer a practical computational alternative to fully fine-tuned transformer models because SBERT is used as a pre-trained embedding component rather than through end-to-end fine-tuning; however, computational efficiency was not directly benchmarked in this study. It should be noted, however, that BERT also achieved higher precision than the hybrid model (0.9444 vs. 0.8333), meaning it produced comparatively fewer false-positive ambiguity flags; the hybrid model's advantage is therefore concentrated in recall rather than uniformly across all metrics, and the choice between the two would depend on whether an application prioritizes minimizing missed ambiguous requirements or minimizing unnecessary review effort.

Although transformer-based architectures such as BERT and RoBERTa have demonstrated strong performance in various NLP classification tasks, the experimental results indicate that ambiguity detection in software requirements benefits from the integration of lexical, structural, and contextual information. While BERT provides powerful semantic representations, the proposed hybrid framework combines complementary linguistic signals that are particularly relevant for identifying ambiguous requirement statements. Consequently, the hybrid model achieved competitive and more balanced performance across both datasets. Its use of pre-trained SBERT embeddings rather than end-to-end transformer fine-tuning suggests a potentially simpler computational setup, although direct computational comparisons were not performed. The scope of this comparison should nevertheless be acknowledged. Fine-tuned BERT was evaluated only on the SRS dataset; therefore, no direct conclusion can be drawn regarding the relative performance of the hybrid framework and BERT under the stronger class imbalance present in FR-NFR. The current transformer comparison is intended as a dataset-specific contextual baseline rather than a comprehensive state-of-the-art benchmark. Extending the same evaluation protocol to FR-NFR and comparing the proposed framework with additional transformer architectures remain important directions for future work.

The transformer baseline evaluated in this study is a fine-tuned encoder-only BERT model; it does not extend to more recent generative and instruction-tuned large language models (LLMs) such as GPT-family models, nor to retrieval-augmented or domain-adapted transformer variants that have since become common in NLP4RE research. Evidence from adjacent studies suggests that instruction-tuned LLMs can perform ambiguity-related tasks in zero- or few-shot settings without task-specific fine-tuning, which would make them attractive as a lower-effort baseline, but also that their outputs can be sensitive to prompt design and less consistent across repeated runs than a fine-tuned discriminative classifier. A systematic comparison against GPT-based, instruction-tuned, and retrieval-augmented approaches was beyond the computational scope of the present study, given the API costs and prompt-engineering effort involved, but is acknowledged as an important direction for future work and is revisited in the Conclusion and Future Work section.

### 4.5 Statistical significance results

The Wilcoxon signed-rank test was applied to compare the fold-level F1-scores of the proposed Hybrid SVM model with the TF-IDF SVM baseline. For the SRS dataset, the test produced a *p*-value of 0.3125, while for the FR-NFR dataset, the p-value was 0.1562. Since both *p*-values exceeded 0.05, the null hypothesis of no systematic difference between the compared fold-level F1-scores was not rejected. Therefore, the observed performance gains should be interpreted as empirical rather than statistically established improvements.

Although the proposed hybrid model achieved competitive F1-scores and improved recall in several experimental configurations, the Wilcoxon signed-rank test did not identify statistically significant improvements over the baseline model at the 0.05 significance level. These findings indicate an observed empirical improvement in recall and F1-score in several configurations; however, the available statistical evidence is insufficient to establish that this improvement reflects a consistent population-level advantage over the baseline. Additional validation using larger datasets, repeated experimental trials, and more independent observations is required to determine whether the observed difference is statistically reliable.

### 4.6 Feature-level analysis

This section analyses the contribution of lexical, structural, and semantic representations to ambiguity detection. The objective is to understand how individual and combined feature types influence classification performance before full hybrid integration.

TF-IDF serves as the lexical baseline. Across both datasets, TF-IDF-based models achieve reasonable accuracy but show clear limitations in recall, particularly for Logistic Regression and Random Forest. Although SVM performs comparatively better under TF-IDF, lexical modeling alone fails to detect ambiguous requirements consistently. This indicates that term-frequency information captures surface importance but does not sufficiently model contextual ambiguity.

Character n-gram features introduce structural information by capturing sub-word patterns and vague expressions. In Dataset-1, structural features improve recall compared to weaker lexical configurations, demonstrating sensitivity to ambiguity patterns not visible at the word level. However, in Dataset-2, character-level representations alone remain unstable under severe class imbalance. While structural cues provide useful signals, they are insufficient when used in isolation.

SBERT embeddings incorporate contextual semantic information. In Dataset-1, semantic modeling produces moderate improvements over weaker lexical baselines but does not consistently outperform the strongest TF-IDF configurations. In Dataset-2, SBERT-only models show unstable precision–recall behavior, particularly under imbalance. These results suggest that contextual embeddings improve representation quality but cannot reliably separate ambiguous and clear requirements without complementary lexical or structural cues.

Pairwise feature combinations provide clearer improvements. Both TF-IDF + SBERT and TF-IDF + character n-grams enhance F1-score relative to single-feature models. Structural information appears particularly beneficial when combined with lexical features, while semantic embeddings stabilize lexical signals under certain configurations. Nevertheless, no pairwise combination fully resolves the ambiguity detection challenge across both datasets.

Across the evaluated configurations, the full hybrid representation produced the most favorable recall and F1-score pattern, particularly when combined with SVM. The results suggest that lexical, structural, and contextual representations provide complementary information: TF-IDF captures lexical signals, character n-grams capture structural patterns, and SBERT represents contextual semantics. No single feature type was sufficient to produce equally stable performance across both datasets.

### 4.7 Ablation study on feature contributions

To evaluate the contribution of each feature component, an ablation study was conducted on the SRS dataset using the same Support Vector Machine configuration as the best-performing hybrid model. Individual feature types were systematically removed from the full TF-IDF, character n-gram, and SBERT representation to examine their contribution to recall and F1-score. The corresponding feature-ablation results are presented in Table 8. The ablation analysis was limited to the SRS dataset because it was designed as a focused feature-contribution analysis rather than as a second full experimental evaluation across both datasets. Consequently, the ablation findings should be interpreted as SRS-specific and should not be assumed to establish identical feature contributions for the FR-NFR dataset, where the class distribution is substantially more imbalanced. Future work should replicate the ablation protocol on FR-NFR and additional requirements corpora.

**Table 8:** Feature ablation results on the SRS dataset using the hybrid SVM configuration.

Configuration	Recall	F1-score	Performance impact
TF-IDF + Char N-Gram + SBERT (Full Hybrid)	0.7767	0.8040	Best overall ambiguity detection performance
Without SBERT (TF-IDF + Char N-Gram)	0.7087	0.7644	Reduced contextual semantic understanding
Without Character N-Grams (TF-IDF + SBERT)	0.7379	0.7876	Loss of structural ambiguity sensitivity
Without TF-IDF (Char N-Gram + SBERT)	0.6990	0.7310	Reduced lexical discrimination capability

The SRS ablation analysis indicates that each feature representation contributes differently to ambiguity detection performance. Removing SBERT produced a noticeable reduction in recall and F1-score, indicating the importance of contextual semantic information in identifying implicit ambiguity. Excluding character n-grams reduced sensitivity to structurally vague requirement patterns, while removing TF-IDF weakened lexical discrimination capability. The full hybrid configuration produced the strongest SRS ablation result among the evaluated feature combinations. However, because the ablation was conducted only on SRS, these component-level findings should not be generalized automatically to FR-NFR or other requirements datasets.

### 4.8 Cross-dataset model comparison

This section presents a comparative analysis of the baseline classifiers and the proposed hybrid framework across Dataset-1 (SRS) and Dataset-2 (FR–NFR). The objective is to determine whether hybrid feature integration consistently enhances ambiguity detection performance and to examine how different classifiers behave under class imbalance.

Across both datasets, baseline models trained on single-feature representations exhibit clear limitations. Logistic Regression shows conservative prediction behavior. It achieves high precision but relatively low recall, particularly when trained using TF-IDF features. As a result, a substantial number of ambiguous requirements are misclassified as clear. Random Forest achieves strong accuracy due to its ability to model non-linear patterns; however, its recall remains limited, especially in Dataset-2 where class imbalance is more pronounced. In comparison, Support Vector Machine demonstrates more stable baseline behavior and maintains a better balance between precision and recall even before feature integration.

When hybrid features combining TF-IDF, character n-grams, and SBERT embeddings are introduced, performance improves across all classifiers. Logistic Regression shows a clear increase in recall and F1-score, indicating that feature fusion reduces majority-class bias. Random Forest also benefits from hybrid integration, although it continues to display relatively cautious prediction behavior. In contrast, SVM demonstrates the most consistent and meaningful improvement, achieving the highest F1-scores across both datasets.

The comparison between datasets highlights structural differences that influence model behavior. Dataset-1 contains greater linguistic variation but only moderate class imbalance. Dataset-2 is more structured but exhibits stronger imbalance. Under these conditions, single-feature models become unstable, particularly those relying solely on lexical frequency or semantic embeddings. Hybrid feature integration addresses this issue by combining lexical, structural, and contextual information within a unified representation.

In Dataset-2, the impact of hybrid integration is particularly evident for SVM. Under TF-IDF alone, SVM shows unstable precision. After applying the full hybrid representation, precision increases substantially while recall remains stable, leading to a considerable improvement in F1-score. Logistic Regression also shows notable improvement under hybrid features. Random Forest, however, continues to demonstrate limited recall despite feature fusion.

These findings suggest that the primary source of performance improvement lies in feature engineering rather than increased model complexity. The hybrid representation captures complementary aspects of ambiguity. TF-IDF reflects lexical importance. Character n-grams capture structural patterns. SBERT embeddings model contextual semantics. Since ambiguity in software requirements is inherently multi-dimensional, no single feature type is sufficient in isolation.

The strong performance of SVM can be attributed to its margin-based learning mechanism. The hybrid feature space is high-dimensional and combines sparse and dense representations. SVM is well suited to this type of representation, as it constructs an optimal separating hyperplane that maximizes the margin between classes. Logistic Regression assumes linear separability and is more sensitive to imbalance, while Random Forest aggregates multiple decision trees and tends to favor the majority class. Consequently, both models are less effective than SVM in fully utilizing the fused feature space.

The stronger results achieved by SVM do not indicate a limitation of the framework. Instead, they emphasize the importance of aligning feature design with classifier characteristics. The hybrid representation is specifically designed to model complex ambiguity patterns, and margin-based classifiers are particularly compatible with such feature spaces.

Overall, the cross-model analysis indicates that the hybrid framework provides competitive and comparatively stable empirical performance across the two evaluated datasets. While baseline models may achieve stronger values on isolated metrics, the hybrid SVM configuration provides a favorable recall–precision trade-off and achieves the highest F1-score among the evaluated conventional configurations. These results should, however, be interpreted as empirical observations within the present datasets rather than as statistically established superiority or evidence of broad generalizability.

### 4.9 Robustness and experimental stability analysis

To improve experimental reliability, robustness evaluation was performed using stratified cross-validation and repeated feature-level comparisons. The hybrid framework consistently maintained stable recall and F1-score across folds and datasets, indicating reduced sensitivity to dataset partitioning and imbalance variation. In contrast, single-feature representations exhibited larger performance fluctuations, particularly under severe minority-class imbalance. Furthermore, the consistent performance trends observed across the two datasets provide preliminary evidence of robustness to differences in dataset composition, although dataset-specific effects cannot be excluded.

### 4.10 Error analysis

Error analysis reveals that most false negatives occur in sentences where ambiguity is implicit and context-dependent, such as domain-specific terminology or underspecified constraints. False positives are often associated with sentences containing cue words (e.g., “may,” “fast”) that do not necessarily imply ambiguity in all contexts. These observations indicate that while the hybrid model improves detection, ambiguity interpretation remains dependent on deeper domain knowledge and discourse-level context. For example, the requirement “The system should handle requests efficiently” was misclassified as clear due to the absence of explicit ambiguity markers, although the term “efficiently” introduces vagueness. Similarly, sentences containing domain-specific terminology were occasionally misclassified due to lack of contextual grounding. These findings indicate an observed empirical improvement in recall and F1-score in several configurations; however, the available statistical evidence is insufficient to establish that this improvement reflects a consistent population-level advantage over the baseline. Additional validation using larger datasets, repeated experimental trials, and more independent observations is required to determine whether the observed difference is statistically reliable.

Beyond the specific examples discussed above, recurring sources of misclassification include modal verbs expressing optionality (for example, “may” or “should”) in contexts where the surrounding sentence otherwise reads as a firm obligation, compound statements that bundle an unambiguous functional requirement with a vague non-functional qualifier, and domain-specific terminology whose ambiguity depends on knowledge external to the sentence itself, such as whether a given protocol or component name has a single unambiguous technical meaning within the target system. These categories suggest that further gains are more likely to come from incorporating requirement-level or document-level context than from refining sentence-level lexical or embedding features alone.

### 4.11 Model interpretability

To improve transparency, future extensions of this framework can incorporate explainable AI techniques such as SHAP or LIME to identify feature contributions in ambiguity classification. This would enable stakeholders to understand why a requirement is classified as ambiguous, thereby improving trust and usability in real-world requirements engineering workflows.

For the lexical and structural components of the hybrid representation, SHAP or LIME could attribute individual TF-IDF terms or character n-grams to a given prediction, while attention-weight visualization over the SBERT encoder could highlight which tokens contributed most to the contextual embedding, offering a complementary, model-internal view of semantic ambiguity cues. Combining feature-importance and attention-based explanations would give analysts both a lexical rationale for which words triggered a flag and a contextual rationale for which parts of the sentence the model attended to, which the authors regard as a necessary step before the framework could be considered ready for practitioner-facing deployment.

### 4.12 Threats to validity

Internal validity concerns relate to the possibility that implementation choices, rather than the underlying feature representations, drive the observed results. Hyperparameter settings, including TF-IDF vocabulary size, SVM regularization, and the choice of SBERT model, were fixed *a priori* based on common practice rather than tuned exhaustively for either dataset, which may understate the achievable performance of any single configuration. Pre-processing decisions, particularly the more aggressive normalization applied to TF-IDF and character n-gram features relative to SBERT input, could also asymmetrically influence the comparison between representations. Finally, SMOTE introduces synthetic minority-class instances whose distributional properties do not perfectly match genuine ambiguous requirements, which may inflate recall on synthetic neighbors of real training instances even though SMOTE was applied only within training folds.

Construct validity concerns whether the binary ambiguity label used in this study captures ambiguity as understood in RE practice. The annotation protocol combined explicit linguistic guidelines with manual review, and inter-annotator agreement was assessed on a 200-statement subsample (Cohen's kappa = 0.80), but the underlying construct still reduces a multi-dimensional phenomenon, spanning lexical, syntactic, semantic, pragmatic, and referential ambiguity together with vagueness, to a single binary outcome. This simplification is necessary for supervised classification but may not fully capture the graded, context-dependent nature of ambiguity as experienced by requirements analysts.

External validity is constrained by the reliance on two publicly available datasets rather than proprietary industrial SRS documents, which may differ in vocabulary, formality, and domain specificity from the corpora used here. Both datasets are in English, so the findings may not generalize to other languages or to safety-critical domains with highly regulated requirement-writing conventions. Generalizability across industrial contexts, languages, and application domains therefore remains to be established through future replication on industrial corpora.

Conclusion validity concerns the statistical basis for the study's comparative claims. Stratified five-fold cross-validation and a Wilcoxon signed-rank test were used to assess whether performance differences were consistent and statistically meaningful; the resulting *p*-values, 0.3125 for the SRS dataset and 0.1562 for the FR-NFR dataset, indicate that the hybrid model's improvement over the strongest lexical baseline, while practically useful, was not statistically significant at the 5% level. Fold-to-fold variability, particularly for the Random Forest configurations, further indicates that classifier randomness contributes non-trivially to the reported metrics, and confidence intervals around individual point estimates were not computed in this study.

### 4.13 Limitations of the study

Several limitations should be considered when interpreting the findings of this study. First, the evaluation was conducted on only two publicly available English-language datasets. The SRS dataset is relatively small, with 976 requirement statements, while the FR-NFR dataset contains 6,008 statements but exhibits substantial class imbalance. Although the use of two datasets provides broader empirical evidence than evaluation on a single corpus, it does not establish broad generalizability across industrial, multilingual, safety-critical, or domain-specific requirements.

Second, ambiguity was operationalized as a binary classification task. This simplification does not fully capture the graded and multi-dimensional nature of ambiguity in requirements engineering, including lexical, syntactic, semantic, pragmatic, and referential ambiguity. The framework also operates primarily at the sentence level and therefore cannot fully model ambiguity be arising from relationships or contextual dependencies across multiple requirements.

Third, the fine-tuned BERT baseline was evaluated only on the SRS dataset. Therefore, the transformer comparison should not be interpreted as a complete cross-dataset benchmark or as evidence that the proposed framework consistently outperforms transformer-based approaches. Similarly, the feature ablation study was conducted on SRS only, and its component-level findings require replication on FR-NFR and additional datasets.

Fourth, although the study includes a structured comparison with representative ambiguity detection approaches, direct numerical comparison with all prior studies is constrained by differences in datasets, pre-processing procedures, annotation protocols, class distributions, and evaluation settings. The study also does not provide a comprehensive experimental benchmark against multiple transformer architectures, recent instruction-tuned language models, or retrieval-augmented approaches.

Finally, the statistical analysis was based on only five paired cross-validation folds. The resulting Wilcoxon signed-rank tests therefore have limited power to detect small performance differences, and the non-significant *p*-values should not be interpreted as proof that no underlying difference exists. Larger datasets, repeated experimental trials, additional random seeds, and broader cross-dataset replication are required before stronger conclusions regarding the statistical superiority or generalizability of the proposed framework can be drawn.

### 4.14 Industrial applicability

Beyond its experimental evaluation, the practical value of the proposed framework depends on its computational cost and its fit within existing requirements-engineering workflows. Feature extraction for the hybrid representation is dominated by SBERT inference, which the all-MiniLM-L6-v2 model performs efficiently on commodity hardware; TF-IDF and character n-gram extraction add negligible overhead by comparison. This suggests that the framework could plausibly scale to SRS documents containing several thousand requirement statements, consistent with the size of the FR-NFR dataset used here, although formal throughput and latency benchmarks on production-scale documents were not conducted and are left for future work. In terms of integration, the framework is designed as a screening classifier rather than an autonomous decision-maker: it is intended to flag candidate ambiguous statements for analyst review during requirements inspection, rather than to reject or approve requirements automatically. Such a classifier could in principle be exposed as a batch or API-based service and integrated into requirements management platforms such as IBM DOORS, Polarion, Jama Connect, or Azure DevOps, surfacing predicted ambiguity flags alongside the requirement text during review. Whether practitioners would prefer raw predictions, calibrated probabilities, or explanation-based output is an open question that would benefit from a dedicated usability study with requirements analysts, which is beyond the scope of the present work.

## 5 Conclusion and future work

This study examined ambiguity detection in Software Requirement Specifications using a hybrid NLP framework that integrates TF-IDF, character n-grams, and SBERT embeddings. The results show that combining lexical, structural, and semantic representations provides more balanced and stable ambiguity detection than using individual feature types. The hybrid SVM achieved the strongest practical performance across the evaluated datasets, particularly in F1-score and recall, although the observed improvements over the strongest lexical baseline were not statistically significant at the 5% level. Overall, the findings support hybrid feature integration as an effective approach for capturing the lexical, structural, and contextual dimensions of ambiguity in software requirements. The study is limited to English requirement statements and two publicly available datasets, which may restrict generalizability to larger industrial and multilingual requirements. The framework also operates primarily at the sentence level and does not capture ambiguity arising from relationships or contextual dependencies across multiple requirements. Future work will evaluate larger, multilingual, and industrial requirements datasets. Further studies will explore document-level context, advanced transformer models, and explainable AI techniques such as SHAP and LIME.

## Data Availability

The original contributions presented in the study are included in the article/supplementary material, further inquiries can be directed to the corresponding author.
